# Comparative Evaluation of Pleural Fluid Cholesterol and Total Protein Versus Light's Criteria in Differentiation of Exudates and Transudates: A Cross‐Sectional Analysis at Tribhuvan University Teaching Hospital

**DOI:** 10.1111/crj.70121

**Published:** 2025-08-27

**Authors:** Amrit Raj Subedi, Tritha Man Shrestha, Yagya laxmi Shakya, Bishal Budha

**Affiliations:** ^1^ General Practice & Emergency Physician, Rapti Aadharbhut Hospital Deukhuri‐Dang Nepal; ^2^ Department of General Practice, Maharajgunj Medical Campus Tribhuvan University Teaching Hospital (TUTH) Kathmandu Nepal; ^3^ Bachelor of Medicine, Bachelor of Surgery (MBBS), Maharajgunj Medical Campus Tribhuvan University Teaching Hospital (TUTH) Kathmandu Nepal

**Keywords:** cholesterol, exudates, lights criteria, pleural effusion, protein, transudates

## Abstract

**Introduction:**

Pleural effusions are very common clinical findings in clinical practice. Their proper diagnosis based on different parameters like pleural fluid protein, LDH, ADA, and cholesterol is very important. Light's criteria, though used as a standard method in differentiating pleural effusions as exudates and transudates, usually misidentifies 15%–20% of transudates as exudates. Hence, the role of other criteria like combined pleural fluid cholesterol and total protein in the differentiation of pleural fluid is studied in this study. The objectives of our study are to determine the role of combined pleural fluid cholesterol and total protein in the differentiation of exudates and transudates in cases presenting in Tribhuvan University Teaching Hospital.

**Methods:**

This is a single‐centered, analytical, cross‐sectional, observational study. This is carried out among patients who are diagnosed as a case of pleural effusions from the emergency ward and respiratory ward of Tribhuvan University Teaching Hospital. Data collection was started after ethical approval was taken from IRB. The data were analyzed through SPSS version 22 for data analysis.

**Results:**

Total of 90 patients with pleural effusion were enrolled in this study. Out of 90 patients, 49 patients were male, and 41 patients were female. The mean age of patients was 52.86 (±17.90) years. Tuberculosis was the most common disease associated with effusions, followed by pneumonia and malignancy. Based on Light's criteria, 65 (72.2%), pleural effusions were found to be exudates, and 25 were found to be transudates, with sensitivity and specificity of 98.4% and 85.7%, respectively. According to pleural fluid total protein, it classified 62.2% [*n* = 56] of cases as exudates and 37.8% [*n* = 34] of cases as transudates. Sensitivity and specificity were 88.7% and 96.4%, respectively. Based on pleural fluid cholesterol, 64.4% [*n* = 58] of cases were shown as exudates and 35.6% [*n* = 32] of cases as transudates, with sensitivity and specificity of 93.5% and 100%, respectively. Combined pleural total protein and cholesterol, however, classified 57.8% [*n* = 52] of cases as exudates and 42.2% [*n* = 38] of cases as transudates, with sensitivity and specificity of 83.9% and 100%, respectively.

**Conclusion:**

Though Light's criteria remain the gold standard for distinguishing transudates from exudates, combined pleural fluid cholesterol and total protein examination improve diagnostic accuracy in circumstances where the diagnosis on discharge differs from the outcome from Light's criteria.

## Graphical abstract

Combined pleural fluid total protein and cholesterol demonstrated perfect specificity and positive predictive value (PPV), but Light's criteria showed high sensitivity (98.4%). Thus, combination offers superior diagnostic accuracy as compared to Light's criteria alone, with all test statistically significant (*p* < 0.0001).

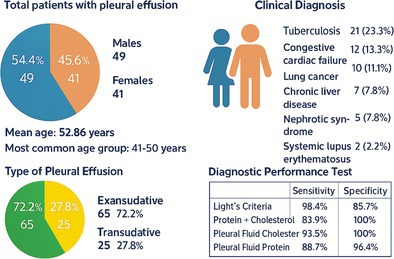

## 1 Introduction

Pleural effusion is defined as a collection of fluid in the pleural space due to increased production or impaired lymphatic absorption [1, 2]. The estimated prevalence of pleural effusion is 320 cases per 100 000 people in industrialized countries, with a distribution of etiologies related to most underlying diseases [3]. Approximately 1.5 million patients experience pleural effusion annually in the United States [4]. However, this prevalence is much higher in the developing world. Unfortunately, there are no studies done extensively in Nepal to determine the prevalence and incidence of pleural effusions. For the treatment of pleural effusions efficiently, it is essential to determine its cause. An integrated step‐by‐step approach that combines clinical assessment, imaging, and pleural fluid analysis is crucial to accurately and efficiently diagnosing pleural effusion [5, 6].

Pleural effusion continues to pose a significant challenge in Nepal. The majority of cases seen in the emergency and outpatient departments are linked to pleural effusions, leading to significant distress, illness, and death among patients. Therefore, promptly diagnosing and distinguishing between these cases is crucial for effectively managing the disease and reducing morbidity. In addition, Light's criteria, it is still used as a gold standard for diagnosing pleural effusions. However, over the last few years, many workers have noted that even Light's criteria misclassify a significant percentage of pleural effusions to diagnose transudates or exudates [7].

**Light's criteria** are used in the differentiation of transudates from exudates as follows. Exudative pleural effusions meet a minimum of one of the following criteria, but transudative pleural effusions meet none:

1. Pleural fluid protein/serum protein > 0.5
2. Pleural fluid LDH/serum LDH > 0.6
3. Pleural fluid LDH > 2/3rd normal upper limit for serum [6].

Light et al. found criteria to have sensitivity and specificity of 99% and 98%, respectively, for differentiating transudative and exudative pleural effusions [8]. Thus, not only are the parameters which we used in Light's criteria for differentiating the effusions effective but pleural cholesterol is also equally effective in diagnosing the effusions as transudative and exudative. At the same time, cholesterol reflects cellular breakdown in exudates, while protein indicates capillary leakage. Pleural fluid cholesterol and total protein offer a simpler, cost‐effective alternative to Light's criteria by eliminating serum testing.

The aim of the study is to determine the role of combined pleural fluid cholesterol and total protein in the differentiation of exudates and transudates in cases presenting at Tribhuvan University Teaching Hospital.

## 2 Methods

### 2.1 Study Design and Setting

This was a single‐center, observational cross‐sectional study done in patients who are admitted to the emergency ward and respiratory ward of Tribhuvan University Teaching Hospital with clinical signs and symptoms suggestive of pleural effusion. The duration of the study was 1 year from August 21, 2021 to August 20, 2022. The study protocol was approved by the Institutional Review Committee of the Institute of Medicine.

### 2.2 Methods

The study included patients with the diagnosis of pleural effusion presenting to the emergency room or admitted to the respiratory ward of Tribhuvan University Teaching Hospital. Patients were excluded if they had pleural effusion secondary to trauma and/or hydropneumothorax.

Sample size for the study was calculated by using the following formula:

n=Zα2PQ/d2

*n* = 95, where *n* = required sample size, Zα = z deviated corresponding to desired reliability level (1.96),

estimated proportion in the population.

Q = 100‐P (where P is prevalence in % and it was 32%) [9].

d = maximum tolerable error (10%).

Using the above information, a sample size of 85 was estimated at a 95% level of confidence and a precision of 10%.

The study began with a brief introduction to the patients and their accompanying relatives, followed by obtaining informed consent for participation. Patients admitted to the emergency and respiratory wards of Tribhuvan University Teaching Hospital, presenting with clinical signs and symptoms indicative of pleural effusion, were evaluated. Routine diagnostic investigations, such as WBC counts and chest X‐rays, were performed for all patients. In cases where chest X‐rays showed minimal or suspected pleural effusion, ultrasonography (USG) of the chest was conducted as necessary.

Thoracentesis was performed on patients confirmed to have pleural effusion. Under aseptic conditions, 10 mL of pleural fluid was collected using a disposable plastic syringe in an inpatient setting. The collected pleural fluid samples were immediately sent to the laboratory for analysis. If immediate transport to the laboratory was not possible, the samples were refrigerated and analyzed within 24 h of collection. The pleural fluid was tested for total protein, lactate dehydrogenase (LDH), glucose, total count (TC), differential count (DC), adenosine deaminase (ADA), Gram staining, and bacterial culture.

Additionally, blood samples were collected and analyzed for parameters including TC, DC, hemoglobin (Hb), erythrocyte sedimentation rate (ESR), random blood sugar (RBS), total and direct bilirubin, aspartate transaminase (AST), alanine transaminase (ALT), total protein, albumin, and LDH. Both pleural fluid and serum samples were collected and tested within 24 h.

In this study, pleural fluid cholesterol levels ≥ 45 mg/dL were used as a specific threshold to classify effusions as exudative [10]. The statistical significance of various parameters included in Light's criteria was assessed and compared with the discharge diagnosis to evaluate their effectiveness in categorizing pleural effusions. The results for pleural fluid total protein, pleural fluid LDH, and pleural fluid cholesterol were recorded and analyzed for each patient to determine their diagnostic accuracy.

Light's criteria and pleural fluid cholesterol were then utilized to classify exudates and transudates, as well as the diagnosis on discharge, using SPSS 22.0 and Microsoft Excel 2016.

## 3 Results

A total of 90 patients with pleural effusion were included in the study. Among them, 49 (54.4%) were males and 41 (45.6%) were females. The mean age of the study population was 52.86% with a standard error of ±17.90 years. The most common age was 41 to 50 years, accounting for 27.8% of the cases, followed by the 31 to 40 years' age group, which comprised 16.7% of the participants (Table 1).

**TABLE 1:** Baseline demographic characteristics of study participants, *n* = 90.

Variables	Frequency (*n*)	Percent (%)
**Sex**		
Female	41	45.6
Male	49	54.4
30 and below	9	10.0
31–40	15	16.7
41–50	25	27.8
51–60	12	13.3
61–70	12	13.3
71–80	8	8.9
81 and above	9	10.0

*Note:* Age mean (SD): 52.86 (±17.90).

In terms of clinical diagnosis, the most common cause of pleural effusion was tuberculosis, found in 21 patients (23.3%), followed by congestive cardiac failure (13.3%), lung cancer (11.1%), and chronic liver disease and nephrotic syndrome (each 7.8%). Less frequently observed conditions included empyema thoracis (5.6%), chronic kidney disease (3.3%), and systemic lupus erythematosus (2.2%) (Table 2).

**TABLE 2:** Disease frequency, *n* = 90.

Disease	Frequency	Percentage
Lung cancer	10	11.1%
Congestive cardiac failure (CCF)	12	13.3%
Chronic kidney disease (CKD)	3	3.3%
Chronic liver disease (CLD)	7	7.8%
Empyema thoracis	5	5.6%
Nephrotic syndrome	7	7.8%
Pneumonia	21	23.3%
Systemic lupus erythematosus (SLE)	2	2.2%
Tuberculosis	23	25.6%
Total	90	100%

When pleural effusion was classified using Light's criteria, 65 cases (72.2%) were categorized as exudate and 25 cases (27.8%) as transudate, as illustrated in Table 3. When compared with clinical diagnosis, Light's criteria identified 61 out of 62 exudates correctly, resulting in a sensitivity of 98.4%. It also correctly identified 24 out of 28 transudates, giving a specificity of 85.7%. The positive predictive value (PPV) was 93.8%, and the negative predictive value (NPV) was 96.0%, all with statistically significant *p*‐values (*p* < 0.0001).

**TABLE 3:** Effusion based on lights criteria, *n* = 90.

		Clinical diagnosis		
		Exudate	Transudate	Total
Light's criteria	Exudate	61 (98.4%)	4 (14.3%)	65 (72.2%)
	Transudate	1 (1.6%)	24 (85.7%)	25 (27.8%)
Total		62 (100%)	28 (100%)	90 (100%)

When using the combination of pleural fluid total protein and cholesterol to classify the effusions, as shown in Table 4, 52 cases (57.8%) were identified as exudates and 38 cases (42.2%) as transudates. This method showed a sensitivity of 83.9% for exudates and a specificity of 100% for transudates. Notably, all 28 transudates were correctly identified, with no false positives. The statistical analysis showed a highly significant correlation between this method and the clinical diagnosis, with a *p*‐value < 0.0001.

**TABLE 4:** Effusion types based on combined pleural fluid cholesterol and total protein, *n* = 90.

		Clinical diagnosis			
		Exudate	Transudate	Total	*P*‐value^1^
Pleural fluid total protein and cholesterol	Exudative	52 (83.9%)	0%	52 (57.8%)	< 0.0001*
	Transudative	10 (16.1%)	28 (100%)	38 (42.2%)	
Total		62 (100%)	28 (100%)	90 (100%)	

^1^ denotes for chi‐square test.

* denotes for statistically significant at *p* < 0.05.

Further analysis showed that pleural fluid cholesterol alone had a sensitivity of 93.5%, a specificity of 100%, a PPV of 100%, and an NPV of 87.5%, making it the most specific marker among the three methods, as illustrated in Table 5. Pleural fluid total protein alone also demonstrated good diagnostic performance, with a sensitivity of 88.7%, a specificity of 96.4%, a PPV of 98.2%, and an NPV of 79.4%.

**TABLE 5:** Sensitivity and specificity of Light's criteria and pleural fluid total protein and cholesterol, *n* = 90.

Parameters	Sensitivity	Specificity	PPV	NPV	*P*‐value^1^
Light's criteria	98.4%	85.7%	93.8%	96.0%	< 0.0001*
Pleural fluid protein	88.7%	96.4%	98.2%	79.4%	< 0.0001*
Pleural fluid cholesterol	93.5%	100%	100%	87.5%	< 0.0001*

^1^ denotes for chi‐square test.

* denotes for statistically significant at *p* < 0.05.

In summary, while Light's criteria remained the most sensitive for detecting exudates, pleural fluid cholesterol demonstrated superior specificity. The combined use of pleural fluid cholesterol and total protein offered a highly specific and statistically significant adjunct or alternative to Light's criteria for the differentiation of exudative and transudative pleural effusion.

In our study, maximum cases were of 41–50 years' age group (27.80%) followed by 31–40 years age group (16.7%). The mean age group of the patients was 52.86 (±17.90) years.

## 4 Discussion

In our study, 90 individuals were considered, 28 with transudative and 62 with exudative pleural effusion. There were 49 males and 41 females. Pleural effusion (PE) was most prevalent in the age group 41–50 years. The patients had a mean age of 52.86 (17.90) years, comparable to the study done by Liam et al., who reported a mean age of 51.2 (±19.2) years [7].

The most frequently encountered cause of exudative PE was tuberculosis (25.6%) followed by parapneumonic effusion (23.3%) and lung malignancy (11.1%) which was similar to Liam et al.'s study in Malaysia, where tuberculosis accounted for 44.1% of cases [7].

In our study, Light's criteria exhibited sensitivity of 98.4% and specificity of 85.7%, with a PPV of 93.8% and a NPV of 96.0% (*P*‐value < 0.0001), which closely aligns with a study carried out by Burgess et al. at Tygerberg Hospital, South Africa, which reported sensitivity of 98% and specificity of 83% among 500 PE cases [11].

For pleural fluid total protein (> 3 g/dL) for exudates, out of 62 pleural exudative effusions confirmed by discharge diagnosis, 55 were correctly classified as exudates and seven were misclassified as transudates. Out of 28 transudates by final diagnosis, 27 were correctly diagnosed as transudates, and one was misclassified as an exudate. This criterion had a sensitivity, specificity, PPV, and NPV of 88.7%, 96.4%, 98.2%, and 79.4%, respectively, with a significant *p*‐value of < 0.0001.

Based on pleural fluid cholesterol, our study showed 64.4% [*n* = 58] of cases as exudates and 35.6% [*n* = 32] of cases as transudates. All 28 transudates were correctly classified, while four exudates were misclassified as transudates. This resulted in a sensitivity and specificity of 93.5% and 100%, respectively. The result was similar to the study carried out by Guleria et al., which showed pleural fluid cholesterol had a sensitivity of 88% and a specificity of 100% [12]. However, it contradicts the findings of Rufino et al. performed in the Pulmonology Department of State University of Rio de Janeiro, which reported a sensitivity of 97.22%, a specificity of 85.71%, a PPV of 98.59%, and a NPV of 75% [13]. This variation may be due to the different threshold for pleural fluid cholesterol (as they used ≥ 50 mg/dL), study settings, population, and locations. Additionally, the study done by Hamal et al. also reported a sensitivity of 97.7% and specificity of 100% for differentiating exudative and transudative pleural effusion, aligning closely with our present study [10].

In our study, combined pleural fluid cholesterol and total protein identified 57.8% [*n* = 52] of exudates but misclassified 10 exudates as transudates. Among 42.2% [*n* = 38] identified transudates, all 28 transudates were correctly confirmed as transudates, giving sensitivity and specificity of 83.9% and 100%, respectively. The results were consistent with the findings of Bist et al.'s study, which reported an impressive sensitivity of 96.6% and perfect specificity of 100% [14]. This study revealed that pleural fluid cholesterol alone and in combination with total protein offered greater specificity in distinguishing exudates from transudates, surpassing the diagnostic accuracy of Lights's criteria.

## 5 Conclusion

Light's criteria remain the benchmark for differentiating transudative from exudative pleural effusion. However, using pleural fluid cholesterol and total protein alone provides comparable accuracy while eliminating the need for accompanying blood tests. In resource‐limited settings, combined pleural fluid cholesterol and total protein can be used as an alternative to Light's criteria to avoid the necessity for simultaneous blood samples and simplify the diagnosis procedure.

## Author Contributions

**Amrit Raj Subedi:** study concept, writing and editing of original manuscript. **Tritha Man Shrestha:** manuscript reviewer and supervisor. **Yagya Laxmi Shakya:** manuscript reviewer and supervisor. **Bishal Budha:** data collection, writing and editing of original manuscript.

## Ethics Statement

Ethical clearance was obtained from IRC IOM TUTH (Ref no: 46 [6–11] E2 78/79) before conducting this study, and a written consent was also taken from the department of General Practice and Emergency Medicine, Tribhuvan University Teaching Hospital before data collection.

## Consent

Written informed consent was taken from each participant.

## Conflicts of Interest

The authors declare no conflicts of interest.

## Data Availability

The data that support the findings of this study are available on request from the corresponding author. The data are not publicly available due to privacy or ethical restrictions.
